# Demonstration of high-frequency self-pulsing oscillations in an active silicon micro-ring cavity

**DOI:** 10.1038/s41598-024-75295-3

**Published:** 2024-10-11

**Authors:** Abdou Eltamimy Shetewy, Mircea Traian Catuneanu, Menglong He, Kambiz Jamshidi

**Affiliations:** Integrated Photonic Devices Group, Chair of RF and Photonics engineering, TU Dresden, 01069 Dresden, Germany

**Keywords:** Integrated optics, Nonlinear optics, Nonlinear optics

## Abstract

We experimentally investigated the self-pulsing (SP) oscillations induced by the thermo-optic, free carrier, and Kerr nonlinear effects in integrated active silicon microring resonators. We demonstrate high frequency self-pulsing oscillations (up to 30 MHz) by applying a few millivolts of reverse bias voltage to the PIN junction of the active cavity. We illustrate that the shape of those oscillations (i.e., frequency and duty cycle) can be controlled by adjusting the CW input power and applying a reverse bias voltage to the PIN junction for carrier removal. This controlling is important for synchronizing the cavity which is crucial for neural network applications. Furthermore, we utilize a mathematical model for visualizing the stability regions by numerically studying coupled mode theory in silicon microcavity under different conditions.

## Introduction

Photonic integrated circuits (PICs) play a pivotal role in a wide range of applications, including but not limited to optical communication^1,2^, signal processing^3–6^, and optical and quantum computing^7,8^. Highly integrated optical circuits require the practical realization of ultra-compact optical resonant cavities to realize more energy-efficient devices. Silicon is the most important material in modern technology and industry due to its unique combination of electrical, mechanical, and chemical properties, as well as its affordability and abundance. Silicon microring resonators (MRR) are a perfect candidate for a wide range of applications due to their potential for large optical nonlinearity, compact footprint, narrow wavelength selectivity, and the compatibility of their fabrication with existing complementary metal–oxide–semiconductor (CMOS) technology. By increasing the circulating optical power inside MRR, the resonant wavelength can be shifted to the red side due to thermal effects, resulting in interesting dynamic behaviors. Those behaviors have been represented in nonlinear processes such as optical bistability (BI)^9–11^, self-pulsing (SP)^12,13^, and even chaotic behavior^14^.

**Fig. 1:**
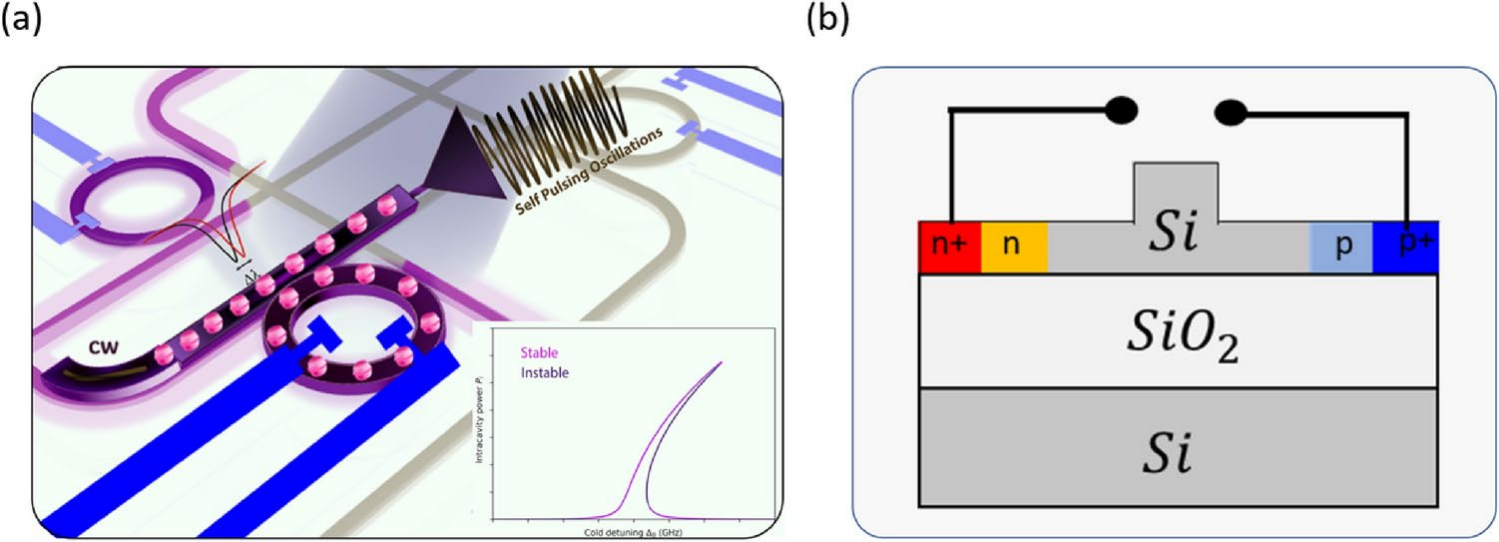
(**a**) Illustration of the system: continuous-wave light enters the cavity and produces high-frequency oscillating output at multiple frequencies. [inset; the bistable curve of a single pump for pulse formation]. (**b**) Cross-section of the device.

Optical SP has been demonstrated theoretically and experimentally in various photonic components such as photonic crystals (PC)^15^ and silicon MRRs by utilizing a dispersive nonlinearity that exhibits a distinct hysteresis response^16^. The concept of SP in a silicon MRR is presented in Fig. 1a, where a continuous wave (CW) laser acts as an input to the resonator and generates a self-sustained oscillation without any external modulation. It occurs when the monochromatic light source has a frequency in the vicinity of the MRR’s resonance and causes a significant increase in the field intensity inside the cavity, which can shift its resonance frequency away from the excitation frequency. Then, the circulating field inside the MRR goes down, and the resonance frequency shifts back to its original position due to the thermo-optic effect and forms a pulse. The circulating power inside the ring versus the frequency detuning as a visualization for pulse formation is illustrated in the inset of Fig. 1a.

Recently, optical SP oscillations-based silicon MRRs become a good candidate for several applications including but not limited to a random number generator and an Ising XY machine^11^, optical communications^17^, optical signal processing^18^, all-optical control and synchronization of the resonator dynamics^19^, and sensing applications^20^. Furthermore, numerous applications in the field of advanced neuromorphic circuits have been implemented using specifically SP-based MRR in different network configurations^21–24^. As reported in^24^, not all optics-based implementation artificial neural networks (ANNs) need to employ neuron “spiking”. Yet, with the prediction and control of self-pulsing oscillation regimes, one can emulate typical neuron-like features, such as refractory periods, temporal integration, and inhibitory behavior in complex coupled networks. Self-pulsing period and spiking time series synchronization of MRR-based optical neurons have also been recently studied in^23^. This is achieved by feedback loops with tunable optical delay and coupling.

In this work, we study SP in active MRR and observed high self-pulsing oscillations (up to 30 MHz) with an input power of 2.588 mW inside the waveguide bus and 100 mV reverse-biased voltage applied on the PIN junction. Additionally, we studied the SP oscillations in this MRR at different conditions, i.e., different bias conditions, CW input powers, and cold detuning. We controlled the shape (i.e., frequency, duty cycle, and distortion) of SP oscillations by adjusting the CW input power. Moreover, we utilize a mathematical model to describe those oscillations’ behavior and BI stability regions. This article is organized as follows. “Experimental setup and device characterization” section presents the experimental setup and device characterization for the self-pulsing mechanism using a CW-pumped laser. SP oscillations are observed in “Self-pulsing observation at different conditions” section including the thermo-optic effect and other physical effects. The discussion and mathematical model are presented in “Mathematical model” section. The conclusion is presented in “Conclusion” section.

## Mathematical model

Light propagation in silicon MRRs is described by nonlinear coupled mode theory (CMT). In this work, we modeled a high-frequency SP in the silicon MRR by using the following set (Eqs. 1, 2, 3) of normalized coupled ordinary differential equations (ODEs), modified from current research on BI, SP, and thermo-optic multistability^6,11,16,25–30^. In this model, the set of ODEs is written in terms of normalized time ($\tau$), normalized intracavity field (*a*), normalized free-carrier density (*n*), and normalized temperature (*T*) quantities, as follows:

1 $$\begin{aligned} \begin{aligned} \frac{\partial a}{\partial \tau } =&-\overset{Linear}{\overbrace{(i{\delta } + 1 + \eta \frac{\partial ^2 {a}}{\partial {t}^2}) a}} - \overset{FCD/FCA}{ \overbrace{\left( i+\mu ^{-1}\right) {n}_c a}} - \overset{Kerr/TPA}{\overbrace{(r-i)|{a}|^2 a}}+ \overset{Thermal}{\overbrace{iTa}} + {S}_{\textrm{in}}\end{aligned}, \end{aligned}$$

2 $$\begin{aligned} \begin{aligned} \frac{\partial {n}_{c}}{\partial {\tau }}&= \frac{1}{{\tau }_{c}}(\bar{\chi }_{c}\left| {a}\right| ^{4}- {{n}_{c}}),\end{aligned} \end{aligned}$$

3 $$\begin{aligned} \begin{aligned} \frac{\partial {T}}{\partial {\tau }}&= {\bar{\chi }}_{th}(\phi \left| {a}\right| ^{4} + \frac{{n}_{c}}{\mu r}\left| {a}\right| ^{2} + \frac{\phi \tau _{ph}v_{g}\alpha _{abs}}{r}\left| {a}\right| ^{2}) - \frac{\Delta {T}}{{\tau }_{th}},\end{aligned} \end{aligned}$$

where $|a|^2$ is the normalized energy inside the ring, $\tau$ is normalized to the photon lifetime; $\tau = t/2\tau _{\textrm{ph}}$ with $\tau _{\textrm{ph}}=t_R /(\alpha L+\theta )$, $\alpha$ is the total linear loss inside the cavity, *L* is the cavity length, $t_{R}$ is the round-trip time, $\theta$ is related to the coupling loss, $\delta$ is the normalized input light frequency detuning, $\eta$ takes $-1$ or $+1$ values to define the sign of the waveguide group-velocity dispersion, see Fig. 1 in Supplementary Note 1. $n_{c}$ is the normalized free-carrier, $\mu$ and *r* are dimensionless quantities related to the ratio of dispersive to absorptive nonlinearity. $S_{in}$ is the normalized input field. $\tau _{c}$ is the normalized carrier lifetime, ${\bar{\chi }}_c$ is the free-carrier dispersion (FCD) term, $\bar{\chi }_{th}$ is the normalized thermal nonlinear coefficients, $\phi$ is the dissipation-per-absorption parameter; it can be adjusted by applying reverse voltage, $\alpha _{abs}$ is the linear absorption loss, $v_{g}$ is group velocity (related by the cavity round-trip time $v_{g} = L/t_{R}$), and $\tau _{th}$ is the normalized normal decay time, $\Delta T$ is the thermo-optic cavity detunings. $\tau _{c}$ and $\bar{\chi }_c$ can be tuned by the reverse bias voltage; see Supplementary Note 2 for details.

**Fig. 2:**
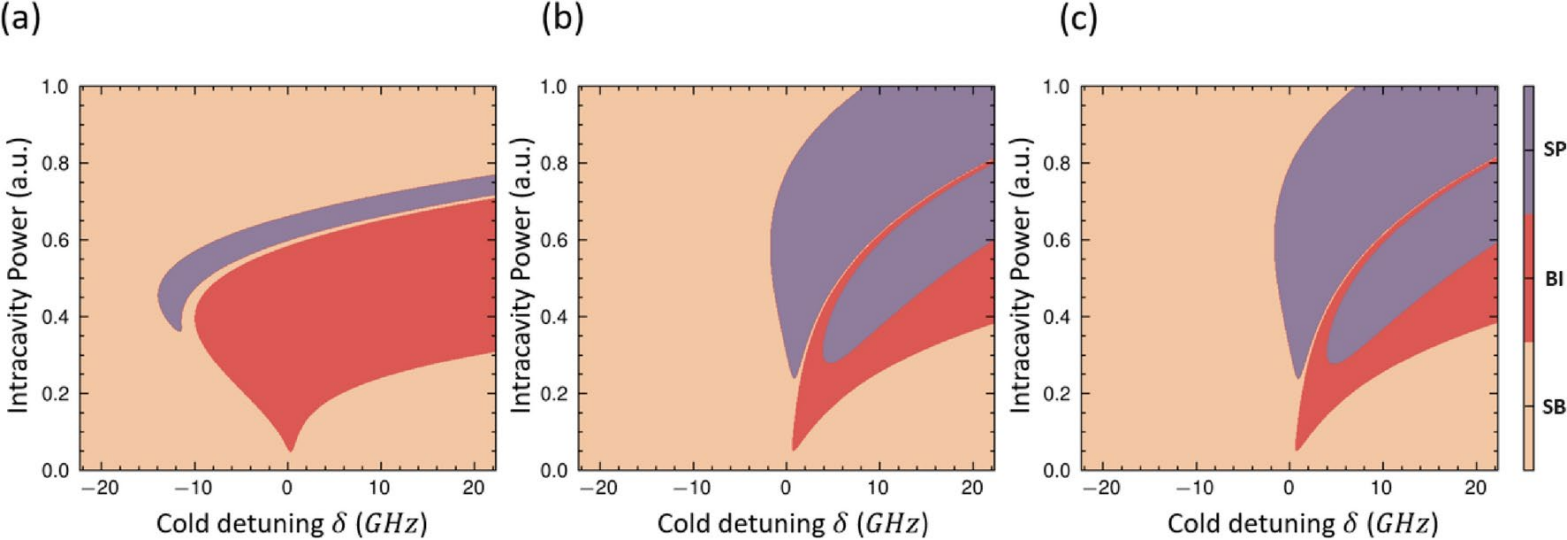
Illustration of universal dynamics map with various intracavity power and detuning for three cases. (**a**) Without applying voltage. (**b**) At 0 V. (**c**) At 100 mV reverse bias. Where SB refers to the optical stable regime, SP and BI are the instability (Self-pulsing) and bistability regimes, respectively.

To investigate the boundaries and regimes of the dynamics including thermo-optic induced self-pulsing (SP), we have applied the linear stability analysis in three steps to the governing equations including Eqs. (1), (2), (3) in the active silicon microcavities. As a first step, we analyze the equilibrium points of the optical field (a), free carrier number ($n_{c}$), and temperature (*T*) in the microring at the steady states (i.e., by letting $\frac{\partial a}{\partial \tau } = 0$, $\frac{\partial {n}_{c}}{\partial {\tau }} = 0$ and $\frac{\partial {T}}{\partial {\tau }} = 0$). We can obtain a system of coupled polynomial equations by substituting this first-order derivative as zero. Next, we have linearized the coupled mode equations and derived the Jacobian matrix, which contains four variables as *a*, $a^{*}$, $n_{c}$, and *T* in the system. Finally, the stability analysis can be obtained according to the three possible cases of the eigenvalues. stability (SB) can be obtained when all eigenvalues have a negative real part. optical BI is obtained when only one of the eigenvalues has a positive real part. For the optical SP, the Jacobian of the system has two pairs of conjugated eigenvalues, which means the number of positive real-part eigenvalues is larger than one. Moreover, we have numerically simulated the eigenvalues by varying the pump detuning and pump power with and without reverse bias voltage. As a result, we have constructed the universal dynamics map with various intracavity power and detuning in Fig. 2 to characterize the condition of the optical stable regime, bistability, and instability, i.e., refer to the optical SP region. It can be concluded that the power threshold of the instability regime is larger than stable one. Notably, we have summarized the effect of applying voltage as 0 V and 0.1 V on the dynamic map. First, by applying reverse bias, the thermo-optic effect has been significantly enhanced (due to resistive heating), and the instability mainly exists on the red side due to the thermo-optic effect. Secondly, the instability is more significant than bistability in the high-power region with reverse bias. In particular, the possible intracavity power can be obtained through the optical bistable curves. We can conclude that the regimes of self-pulsing have been dynamically enlarged by increased pump power and reverse bias voltage.

The borders of both BI and SP as functions in cavity circulating power and cold detuning are examined and presented in Fig. 2a–c for no applying voltage, 0 V, 100 mV reverse bias, respectively. From the experimental analysis presented in “Self-pulsing observation at different conditions” section, we deduce how the input light parameters (i.e., power and wavelength) and biased conditions affect the boundaries of SP. The shape and dynamics of the SP oscillation can be controlled by controlling the input power levels inside coupling bus waveguides, detuning, and applied reverse bias voltage (i.e., free carrier lifetime).

## Experimental setup and device characterization

The device under test (DUT) is an MRR with a radius of 20 $\mu$m coupled to a bus waveguide with a PIN junction, which is also embedded in the ring waveguide and terminated with a two-port electrode, as illustrated in Fig. 1a, thus making it possible to control the carrier density and carrier lifetime in the ring waveguide by applying external reverse biased voltage. This MRR has been fabricated by a multi-project wafer service at a CMOS foundry (IMEC, Belgium) run on an SOI wafer of 220 nm thickness. The ring uses rib waveguides with an inner core height of 220 nm, an outside slab height of 60 nm, and a core width of 450 nm, where this waveguide can only support fundamental transverse electric $TE_{00}$ and transverse magnetic $TM_{00}$ modes. Figure 1b presents the cross-section with four different doping levels, where those doping concentrations inside the waveguide’s sections (i.e., $n, n^{+}, p,$ and $p^{+}$) are performed by doping levels of IMEC.

**Fig. 3:**
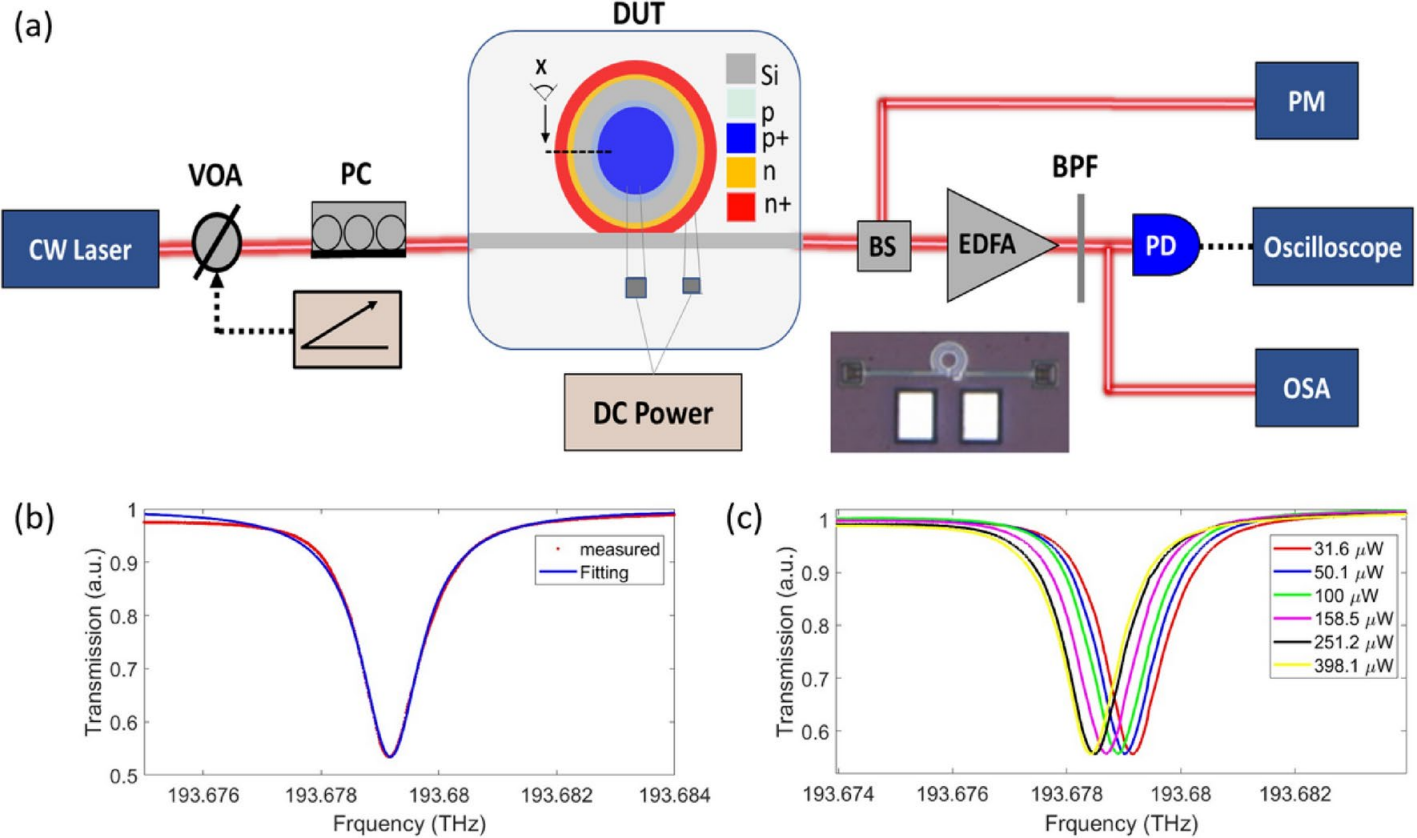
(**a**) Schematic of the experimental setup; The CW laser is attenuated by a Variable Optical Attenuator (derived by triangle electric signal) to adjust the light power and then is polarized by a polarization controller, the polarized light is coupled to the MRR within the input grating coupler. A DC power is used to adjust the free-carrier lifetime inside the cavity. *CW laser* tunable continuous-wave laser, *VOA* variable optical attenuator, *PC*, polarization controller, *DUT* devise under test, *BS* Beam splitter, *EDFA* erbium-doped fiber amplifier, *BPF* band pass filter, *PD* Photodetector, *PM* power meter, *OSC* Oscilloscope. Solid red lines show optical fibers and dashed lines show microwave cables. (Insert; Microscopic image of the fabricated device). (**b**) Normalized output transmission spectrum for a typical $TE_{00}$ resonance profile with a Lorentzian fit, showing $\kappa _{00}/2\pi = 1.06$ GHz. (**c**) Measured optical transmission of the silicon MRR at various input power levels; the quoted input power refers to the power at the bus waveguide before coupling into the MRR.

Figure 3a depicts a schematic of the measuring setup. A DC voltage source controls a variable optical attenuator (THOLABS V1550A U02033) to adjust the light power of a tunable continuous-wave laser (Toptica DLC Pro), which is then polarized by a polarization controller (THOLABS FPC561), and the polarized light is coupled into the MRR with the input grating coupler. The light collected from the output grating is fiber-coupled and split into two parts by a 99:1 beam splitter (THOLABS TW1550R1A1), one part is fed into an optical power meter (Thorlabs PM100USB) for power measurement and alignment check while the other is amplified by an erbium-doped fiber amplifier (Thorlabs 1550 Fiber Amplifier) and filtered by a tunable band-pass filter centered at the laser wavelength to remove the spontaneous emission of the amplifier, and increasing the signal-to-noise ratio (SNR). To test the real-time waveforms, the amplified signal is fed into a real-time oscilloscope (KEYSIGHT MXR 604A) via a fiber-based photodetector (THOLABS DET08CFC). Throughout the experiment, a thermoelectric heater is used as a common substrate for the chip to improve temperature stability and reduce the sensitivity of the chip temperature to surrounding/environment temperatures. Using this heater, the resonance wavelength became more stable, therefore the SP operation was more stable and was reproduced effectively.

The linear and nonlinear normalized optical transmission of the DUT at various input powers are presented in Fig. 3b, c, respectively, where the quoted input power refers to the power at the bus waveguide before coupling into the MRR. Figure 3b shows normalized output transmission spectrum for a typical $TE_{00}$ resonance with a Lorentzian fit. For each resonance fit, we can extract the intrinsic loss rate ($\kappa _{00}/2\pi$), external coupling rate ($\kappa _{ex}/2\pi$), and loaded linewidth ($\kappa /2\pi = \kappa _{00}/2\pi + \kappa _{ex}/2\pi$)^31^. This resonance is measured at very low power ($\sim$ 31.6 $\mu$W) inside the bus waveguide, where the MRR at this power level is in a highly linear region. The resonance is under-coupled ($\kappa _{00}>\kappa _{ex}$), with fitted $\kappa _{00}/2\pi = 1.06$ GHz, and we also record a linewidth (i.e., FWHM) of 1.225 GHz, extinction ratio (ER) of 13.5 dB, loaded quality factor of $1.58\times 10^{5}$, and optical propagation loss ($\alpha$) equal to 2.03 dB/cm at the resonant frequency of 193.68 THz.

The effective refractive index of silicon waveguides is dramatically altered by the free-carrier dispersion (FCD) phenomenon, which shifts the resonant wavelength to the blue side (blueshift), as well as the thermo-optic effect which shifts the resonant wavelength to the red side (redshift). Figure 3c represents the normalized output transmission spectrum for a $TE_{00}$ resonance versus frequency in THz at different input power levels in the bus, where the resonant wavelength is red-shifted by increasing the input power. The measurements indicate that, by increasing the input power, the redshift caused by the thermo-optic effect dominates the effect of the blueshift caused by FCD. The power threshold of the nonlinear region is estimated to be around $\sim$ 398.1 $\upmu$W. This power level is incredibly low for a heavily doped silicon MRR, which may expand the silicon MRR’s application space. Minimizing the cross-section and the linear loss for the ring waveguide will effectively decrease the threshold power for the onset of SP and BI.

## Self-pulsing observation at different conditions

Increasing the input power more than the power threshold of the nonlinear region causes the system to exhibit bifurcations, leading to stable optical pulsing states, i.e., self-pulsing. Through our experiment, optical SP is detected when the power inside the waveguide’s bus is raised to around one milliwatt. SP is observed at both positive and negative detunings, predominantly on the red side of the resonance. Various SP regions with different spices (i.e., pulses with different frequencies, ER, and duty cycle) can be reached by controlling the input power levels, detunings, and applied reverse bias (i.e., free carrier lifetime).

**Fig. 4:**
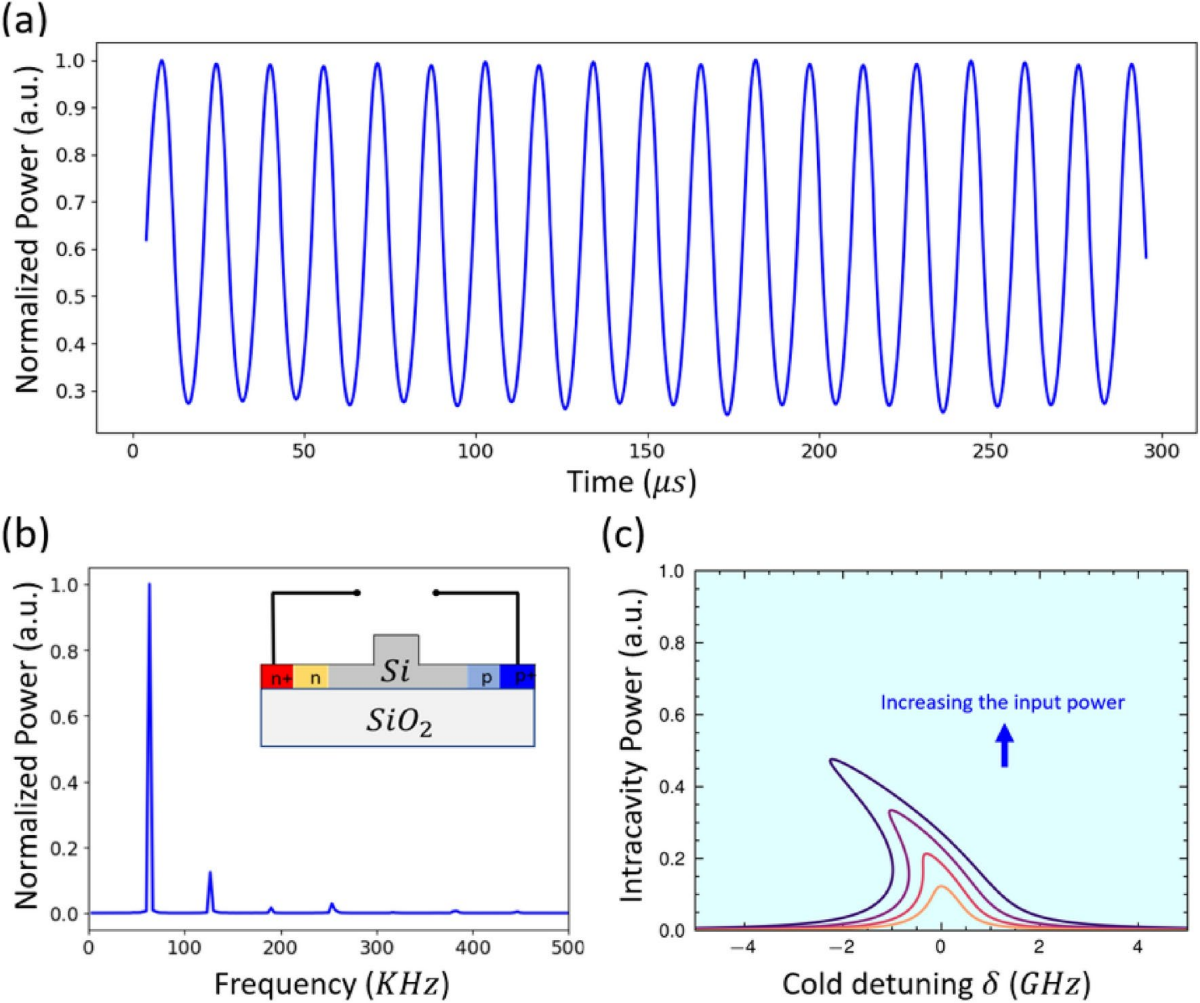
(**a**) Output power in the time domain with input power 1.3 mW and wavelength detuning 3 pm. (**b**) FFT spectrum of the output power. [Insert; the bias voltage on PIN junction]. (**c**) Calculated normalized circulating power inside the ring versus the frequency detuning for different 4 input power (Blue arrow indicates; increasing input power inside the bus). $\tau _{ph}$ = 130 ps, $\tau _{car}$ = 1 ns, $\tau _{th}$ = 30 ns.

Figure 4 presents one example of SP oscillations in a low-frequency region, which appears by applying an input power level of 1.3 mW and a bias-free condition (i.e., without applying voltage on the PIN junction). Figure 4a shows the temporal behavior of the normalized output power oscillates periodically in the time domain (i.e., almost sinusoidal wave behavior), with a high ER and a duty cycle of almost 50%. The fast Fourier transform (FFT) is applied to this temporal output power and depicted in Fig. 4b, the output light field contains one primary frequency close to 67 kHz. Those oscillations are generated from the refractive index changes inside the microring cavity due to the combination of the Kerr, thermo-optic, and FCD nonlinearities. Compared with other effects, the Kerr effect plays a negligible contribution to the resonance shift (the refractive index change). The resonance shift (i.e., the refractive index changes in the ring cavity) is influenced by both thermo-optical and FCD effects. FCD-induced resonance shift, which is the embodiment of carrier density in the ring, and TO-induced resonance shift almost have a comparable contribution. However, FCD-induced resonance shift holds a larger contribution than the TO effect does in some regions, a comparable contribution, and a lower contribution in other regions. To provide an understanding of the mechanism and its fundamental physics, the normalized refractive index changes in the MRR caused by the thermo-optic effect, Kerr effect, and FCD effect were analyzed separately in the time domain^25^. As illustrated in Fig. 4c, we have theoretically investigated the bistable curves of a single pump with different power levels under bias-free voltage conditions. Interestingly, the bending of bistable curves of intracavity power causes the rich dynamics of silicon microresonators, such as bistability and limit cycles. Particularly, the bistable curves bend to the blue side without the reverse bias voltage due to the FCD dominants over the Kerr effect and thermo-optic effect within the medium power range.

**Fig. 5:**
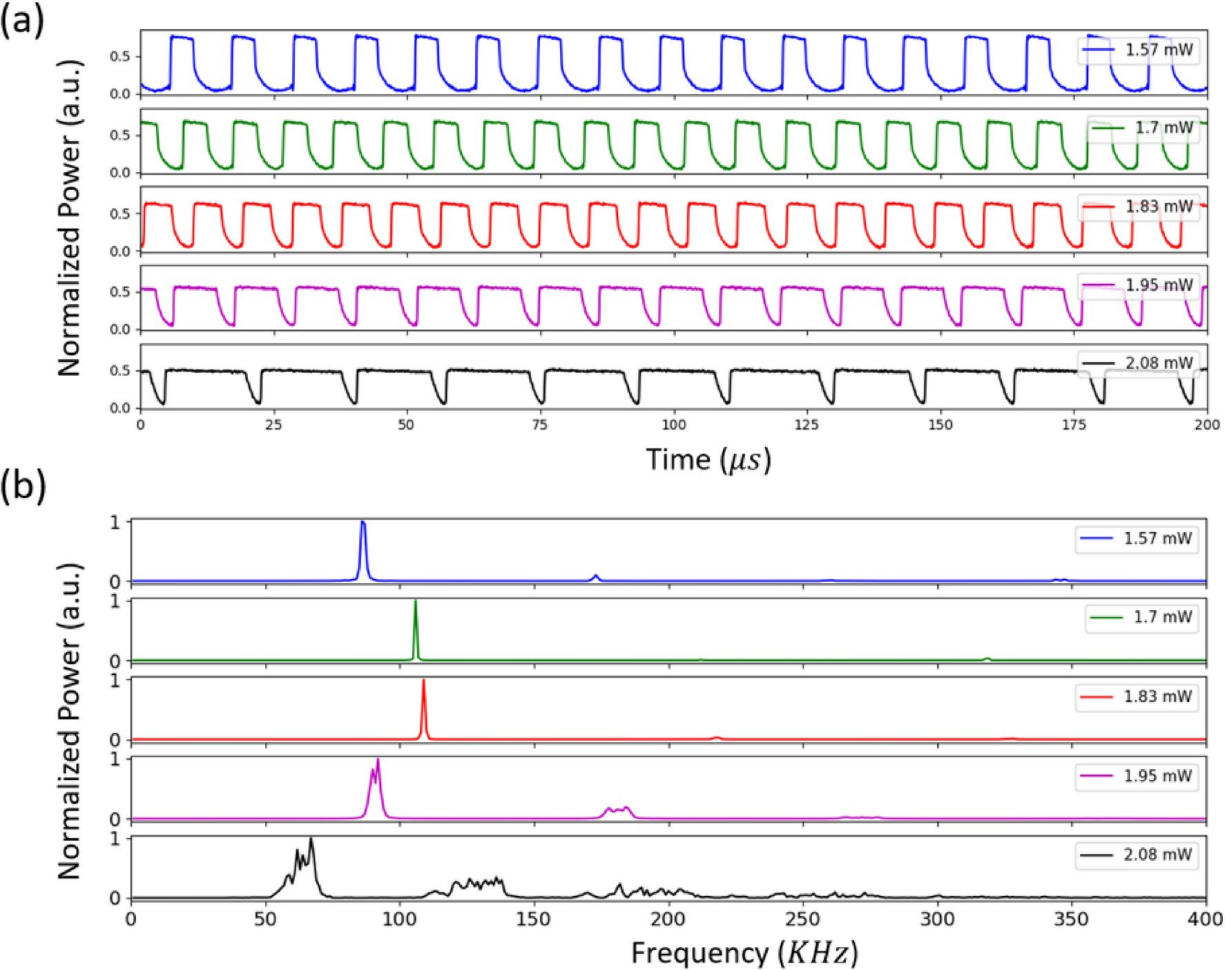
(**a**) Normalized output power in the time domain for five input power levels (ranging from 1.57 mW to 2.08 mW) and fixed wavelength detuning 3 pm. (**b**) FFT spectrum for the five output powers. $\tau _{ph}$ = 130 ps, $\tau _{car}$ = 1 ns, $\tau _{th}$ = 30 ns.

Furthermore, the power dependence of oscillation frequencies is studied. Figure 5a depicts the temporal behavior of the normalized optical transmitted for 5 different SP oscillations at 5 input power levels ranging from 1.57 to 2.08 mW, bias-free, and fixed detuning. As the input power increases, the pulse takes more time for the deformation to recover (return to its original shape or size). This physical phenomenon arises because increased input power causes the microring cavity to warm up faster due to the thermo-optic effect. Additionally, increasing the input power strengthens the free carrier effect inside the cavity. Therefore, both the thermal effect and the free carrier effect cause the microring to deform more drastically. FFT of the 5 SP oscillations is presented in Fig. 5b. At fixed detuning, by increasing the input power, the oscillation’s frequency increases and then decreases back, whereas at high power; oscillations consist of dominant frequency and its harmonics.

**Fig. 6:**
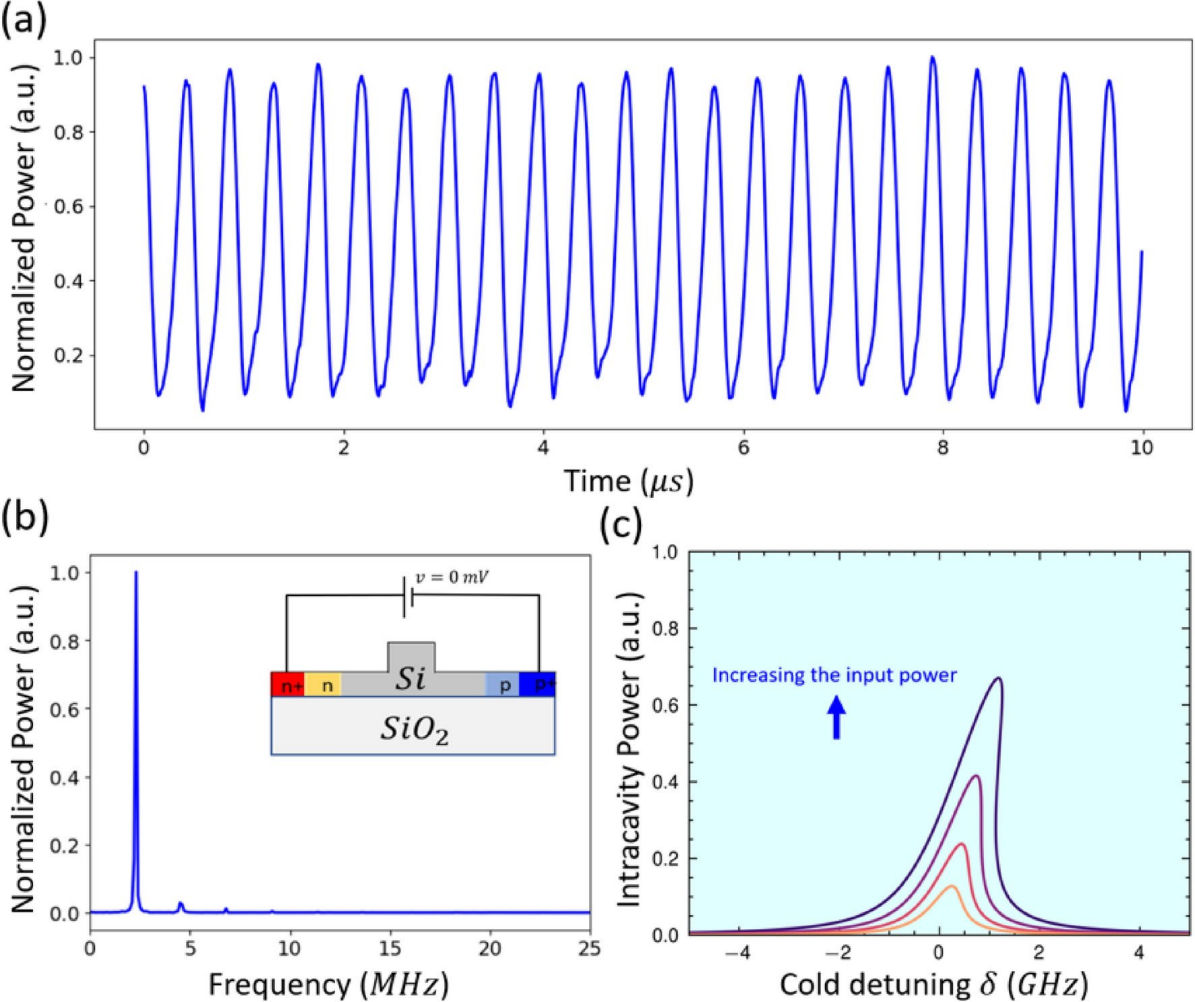
(**a**) Output power in the time domain with input power 2.6 mW and wavelength detuning 3 pm. (**b**) FFT spectrum of the output power. [Insert; applied bias on PIN junction]. (**c**) Calculated normalized circulating power inside the ring versus the frequency detuning for different 4 input power (Blue arrow indicates; increasing input power inside the bus). $\tau _{ph}$ = 130 ps, $\tau _{car}$ = 140 ps, $\tau _{th}$ = 30 ns.

Additionally, the performance of the MRR has been investigated as a function of a free-carrier lifetime when a reverse-biased voltage is applied to the integrated PIN junction, see Supplementary Note 2 and our previous work^28^ for details. Applying reverse bias allows for the continued observation of both optical BI and SP behaviors. However, SP can only be seen when a small applied reverse bias is used; as the applied voltage increases, the SP disappears, leaving just the optical BI. Applying a reverse-biased voltage enables reaching SP regions with high-frequency oscillations. In our most recent study^12^, we found that using a 1 mV reverse bias voltage can produce an SP with multiple frequencies as high as 20 MHz. Figure 6 presents one example of those oscillations at nearly zero voltage applied reverse bias [Insert. of Fig. 6b]. The temporal behavior of the normalized output power oscillates periodically in the time domain, with a high ER and small fluctuation in intensity and duty cycle is presented in Fig. 6a. Those oscillations are observed at an input power level of 2.6 mW. FFT of those temporal oscillations is depicted in Fig. 6b, where the output light field contains one primary frequency close to 2.75 MHz. This oscillation is due to the dominant thermo-optic effect over the FCD effect, where the reverse bias can reduce the free carrier lifetime from 1ns to close or under 140 ps^28^. Figure 6c shows the bistable curves of a single pump with different power levels under 0 V conditions of reverse bias. As the voltage is set to 0 V, the bistability curves have been bent to the red side, indicating that the thermo-optic effect’s strength is larger than that of FCD due to the reduced carrier lifetime. Besides, the intracavity power has increased due to the reduction of the nonlinear loss caused by free carrier absorption (FCA), and the resonance has been shifted to the red side with increasing power.

**Fig. 7:**
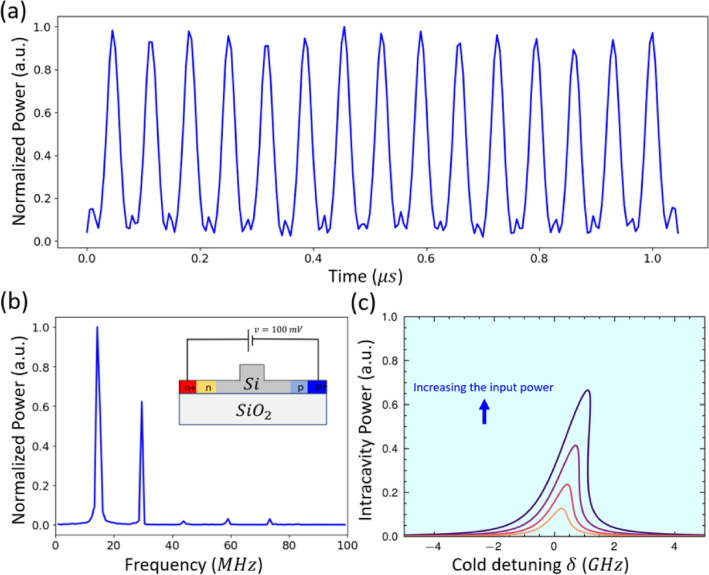
(**a**) Output power in the time domain with input power 1.3 mW and wavelength detuning 3 pm. (**b**) FFT spectrum of the output power. [Insert; the bias configuration on PIN junction]. (**c**) Calculated normalized circulating power inside the ring versus the frequency detuning for different 4 input power (Blue arrow indicates; increasing input power inside the bus). $\tau _{ph}$ = 130 ps, $\tau _{car}$ = 130 ps, $\tau _{th}$ = 30 ns.

Another example of high-frequency SP oscillations is observed at the same input power level (i.e., 2.6 mW) and 100 mV applied reverse bias [Insert. of Fig. 7b]. The output power oscillates in the time domain, with a high extinction ratio and small intensity fluctuation which consequently appear in a semi-random duty cycle (Fig. 7a). The randomness in the duty cycle is observed because intensity fluctuation will result in phase noise and change/randomness in the duty cycle^32^. The FFT presented in Fig. 7b shows that the output light field contains two primary frequencies around 15 MHz and 30 MHz. Those two frequencies are due to the overlap between the thermal and free carrier oscillations, where those oscillations are more sensitive to noise. We believe that the source of the noise in the experiment is optical noise from EDFA or electrical noise from DC probes. However, those intensity fluctuations go up and down with less than 8% of the peak amplitude and will not limit the applications of this oscillation, for instance, in an all-optical spiking in photonic artificial neural networks. Indeed, further investigation is needed to understand the physics of those noises and duty cycle randomness. Figure 7c presents the bistability curves that have been bent more to the red side compared to the case of applying 0V, which indicates the strength of the thermo-optic effect is greater than that of FCD due to the reduced carrier lifetime.

## Conclusion

In conclusion, we experimentally characterized the high frequency SP dynamics in active silicon ring cavities by studying the effects of varying the input optical power and free-carrier lifetime. SP oscillations are controlled by tuning the input power for neuromorphic or optical neuron applications. FCA and FCD are tuned by applying a reverse bias voltage on the PIN junction of the cavity. SP oscillations were still observed with fast oscillations up to 30 MHz using a small voltage bias equal to 100 mV. By increasing the reverse bias voltage more than this amount SP disappeared and BI dominated. Furthermore, We developed a numerical model based on ODEs in active silicon MRR for simulating the bistable curves of a single pump with different power levels under three voltage conditions for reverse bias.

## Supplementary Information

Supplementary Information.

## Data Availability

The datasets used and analysed during the current study are available from the corresponding author upon reasonable request.
