# Supplementary material for: Demonstration of high-frequency self-pulsing oscillations in an active silicon micro-ring cavity
Source: Sci Rep. 2024 Oct 11;14:23823. doi: 10.1038/s41598-024-75295-3 (PMC11470062; doi:10.1038/s41598-024-75295-3)
Supplement: Supplementary file 1 — Supplementary Information. [file 41598_2024_75295_MOESM1_ESM.pdf]

# Supplementary Material: Demonstration of high-frequency self-pulsing oscillations in an active silicon micro-ring cavity

ABDOU ELTAMIMY SHETEWY,[1,*,+] MIRCEA TRAIAN CATUNEANU,[1,*], MENGLONG HE,[1], AND KAMBIZ JAMSHIDI[1,+]

[1]Integrated Photonic Devices Group, Chair of RF and Photonics, TU Dresden, 01069 Germany
[*]These authors contributed equally to the work.
[+]abdou.shetewy@tu-dresden.de / kambiz.jamshidi@tu-dresden.de

## Supplementary Note 1: The design and simulation of microresonator device

MRR in this work consists of a ring waveguide coupled to a straight waveguide (see Fig. 1 in the main text), with a ring radius of 20 $\mu$m and FSR of 615.6 GHz. Both waveguides are rib waveguides with 220 nm height, 450 nm width, and 60 nm slab height, where those waveguides are designed only to support the $TE_{00}$ and $TM_{00}$ modes. The input and output grating couplers are designed to couple and decouple only $TE_{00}$ mode. Figure. 1 illustrate the numerical simulation of this MRR device. The $TE_{00}$ mode field distribution in the microresonator waveguide at a wavelength of 1550 nm is presented in Fig. 1(a). Effective refractive index and group index of the $TE_{00}$mode versus wavelengths shown in Fig. 1(b). This microresonator works in both

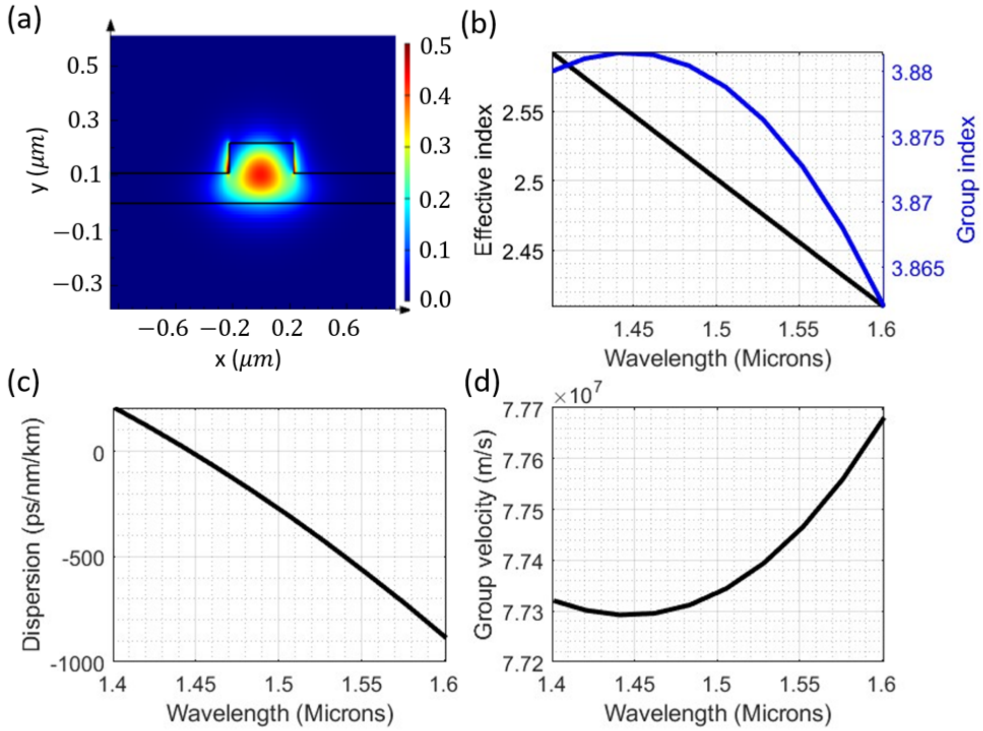


Fig. 1. Numerical simulation of MRR device. (a) $TE_{00}$ field distributions in the 615.6 GHz microresonator waveguide at $\lambda$ = 1550 nm. (b) Effective refractive index and group index of$TE_{00}$ mode versus wavelengths. (c) Dispersion of the $TE_{00}$ versus wavelength. (d) Group velocity of the $TE_{00}$ versus wavelength.

normal and anomalous dispersion regions in the telecom wavelength range, as illustrated in Fig. 1(c). Group velocity versus wavelength is presented in Fig. 1(d).

## Supplementary Note 2: Parameters in normalized ODEs

The terms and coefficients in normalized ODEs are defined as; the normalized energy inside the ring is $|a|^2 = |e|^2/\sqrt{\frac{2\hbar\omega}{\mu r\sigma\gamma v_g \tau_{\mathrm{ph}}\tau_c}}$ where $e$ is the complex amplitude of light propagating in the MRR and $|e|^2$ equal to the $TE_{00}$ mode energy, the Kerr coefficient is $\gamma = 3\omega\,\mathrm{Re}\left[\chi^{(3)}\right]/4\epsilon_0 c^2 n_0^2 = 2\pi n_2/\lambda$; $n_0$ is the effective refractive index, c is the light velocity in a vacuum, $\mathrm{Re}\left[\chi^{(3)}\right]$ is the real part of third order nonlinear optical susceptibility, $\lambda$ is operation wavelength, $n_2$ is Kerr nonlinearity coefficient. $\sigma$ is FCA cross-section, $\delta = 2t_{\mathrm{ph}}\left(\omega_{\mathrm{res}} - \omega\right)$ is the normalized input light frequency detuning, $\omega_{\mathrm{res}}$ and $\omega$ are resonance frequency and input light frequency, respectively. The dimensionless quantities related to the ratio of dispersive to absorptive nonlinearity are defined as $\mu = \frac{(2\omega/c)(-\partial n/\partial n_c)}{\partial\alpha/\partial n_c} = -\frac{4\pi}{\lambda}\frac{\partial n/\partial n_c}{\partial\alpha/\partial n_c}$ and $r = \frac{\mathrm{Im}\left[\chi^{(3)}\right]}{\mathrm{Re}\left[\chi^{(3)}\right]} = \frac{\lambda}{4\pi}\frac{\beta_2}{n_2}$; $\beta_2$ is the TPA coefficient.

Normalized unitless free-carrier $n_c = \frac{\mu\sigma v_g \tau_{\mathrm{ph}}}{2}\bar{n}_c$; $\bar{n}_c$ is carriers in unite of $m^{-3}$. $\bar{\chi}_c = \left(\tau_c/\tau_{\mathrm{ph}}\right)\left(r\mu\sigma_{FCA}/4\hbar\omega\gamma_{\mathrm{Kerr}}v_g\right) \approx 5\tau_c/\tau_{\mathrm{ph}}$ is free-carrier dispersion term. $\bar{\chi}_{\mathrm{th}} = \frac{4\hbar\omega\kappa_\theta}{C_{Si}\mu\sigma}\frac{\tau_{\mathrm{th}}}{\tau_c}$

Table 1. The normalization coefficient values and bulk silicon material properties, as given in Equations (1)–(4), are utilized in the SP modeling.

| Parameter | Value | Units | Source |
|---|---|---|---|
| $n_0$ | 2.454 | — | Simulation |
| $n_g$ | 3.87 | — | Simulation |
| $\sigma_{FCA}$ | $14.5 \times 10^{-22}$ | $m^2$ | Simulation |
| $FSR$ | 615.6 | GHz | Measurement |
| $\alpha$ | 2.4 | dB/cm | Measurement |
| $\alpha_c$ | 0.0017 | — | Measurement |
| $\tau_{th}$ | 30 | ns | Measurement |
| $\tau_{ph}$ | 0.13 | ns | Measurement |
| $t_R$ | 1.62 | ps | Measurement |
| $\hbar\omega$ | 0.8 | eV | Calculation |
| $r$ | 0.189 | — | [1] |
| $\mu$ | 25 | — | [1] |
| $\rho_{Si}$ | $2.329 \times 10^6$ | $g/m^3$ | [2,3] |
| $C_{Si}$ | 0.713 | $J/(gK)$ | [2–4] |
| $\kappa_\theta$ | $1.86\times10^{-4}$ | $K^{-1}$ | [5] |
| $\beta_2$ | $0.75 \times 10^{-11}$ | $m/W$ | [6,7] |
| $n_2$ | $4.5 \times 10^{-18}$ | $m^2/W$ | [6,7] |

is the normalized thermal nonlinear coefficients; $\kappa_\theta = \frac{\partial n}{\partial T}$ is the TO coefficient; $C_{Si}$ is specific heat capacity of silicon. The dissipation-per-absorption parameter is given by $\phi = \frac{qV_{\text{rb}}+2\hbar\omega}{2\hbar\omega}$; $V_{\text{rb}}$ is the reverse applied voltage, q is the electron charge. The TO effect-corresponding normalized temperature change is $T = \frac{\omega_L \kappa_\theta \Delta T}{n_0 \tau_{ph}}$.

Table. 1 provides the normalization coefficients, pertinent device, and bulk silicon material parameters required for the SP model. These parameters are derived from numerical simulation, experimental data, and other work in literature, as described throughout the article.

Free carrier lifetime ($\tau_{fc}$) as a function in applied reverse voltage is modeled in our recent work [8] as $\tau_{\text{fc}} \approx \left(\tau_{\text{fc},0} - \tau_{\text{fc,sat}}\right) e^{-V_{\text{rb}}/V_{\tau_{\text{fc}}}} + \tau_{\text{fc,sat}}$, where $\tau_{\text{fc},0}$ is the maximum Free carrier lifetime (FC-lifetime) when applying 0 V on the PIN junction ($\tau_{\text{fc},0}$ = 140 ps), $\tau_{\text{fc},sat}$ is the minimum FC lifetime limited by velocity saturation ($\tau_{\text{fc},sat}$ = 3.1 ps), $V_{\tau_{fc}}$ is FC-lifetime decay constant equal to 2.15 V. $V_{rb}$ is the applied reverse voltage. The equation illustrates the exponential decay of free carrier lifetime versus applied reverse voltage. A strong enough bias field (around 20 V), can decrease the carrier lifetime from 1 ns to 3.1 ps. FCD parameter ($\bar{\chi}_c$) also decays exponentially as a function in applied reverse voltage.

Free-carrier dispersion is implemented with Drude-model wavelength dependence and illustrated in Fig. 2.a, dispersion increased by increasing wavelength. Effective mode area ($A_{eff}$) is defined as the ratio of a mode's total energy density per unit length to its maximum energy density. Figure. 2.b presents $A_{eff}$ versus wavelength, calculated by the numerical mode solver. The total energy density of the propagating mode per unit length is increased by increasing the operating wavelength. Thermal nonlinearity is sensitive to operating wavelength. Figure. 2.c shows the thermal nonlinear coefficient as a function in wavelength, where the amplitude of this nonlinearity

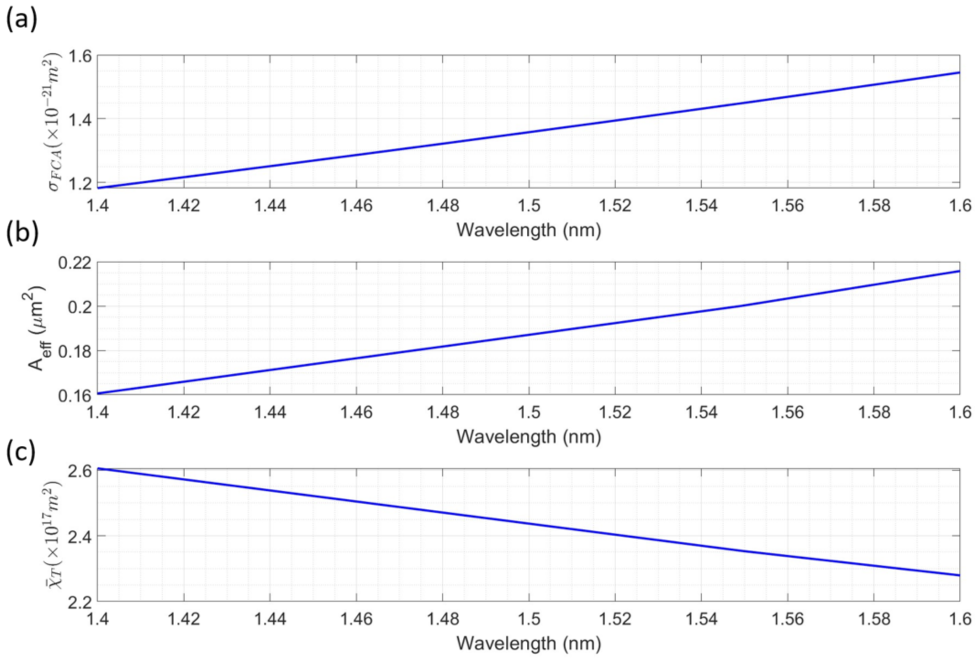


Fig. 2. (a) FCA cross-section versus wavelength. (b) Effective cross-section area versus wavelength. (c) Thermal nonlinear coefficient versus wavelength.

decreases by increasing the wavelength.

## References


1. R. Hamerly, D. Gray, C. Rogers, and K. Jamshidi, "Conditions for parametric and free-carrier oscillation in silicon ring cavities," J. Light. Technol. **36**, 4671–4677 (2018).
2. S. M. Sze and K. K. Ng, "Physics of semiconductor devices," Wiley, New York **3rd ed.** (2006).
3. H. Yuan, Y. Xie, B. Liu, *et al.*, "Self-pulsation and synchronization of optical neurons based on microrings," Opt. & Laser Technol. **172**, 110479 (2024).
4. H. Abe, H. Kato, and T. Baba, "Specific heat capacity measurement of single-crystalline silicon as new reference material," Jpn. J. Appl. Phys. **50**, 11RG01 (2011).
5. G. Cocorullo and I. Rendina, "Thermo-optical modulation at 1.5 mu m in silicon etalon," Electron. Lett. **1**, 83–85 (1992).
6. Q. Lin, O. J. Painter, and G. P. Agrawal, "Nonlinear optical phenomena in silicon waveguides: modeling and applications," Opt. express **15**, 16604–16644 (2007).
7. H. Tsang and Y. Liu, "Nonlinear optical properties of silicon waveguides," Semicond. Sci. Technol. **23**, 064007 (2008).
8. D. Gray, R. Hamerly, M. Namdari, *et al.*, "Thermo-optic multistability and relaxation in silicon microring resonators with lateral diodes," Phys. Rev. Appl. **14**, 024073 (2020).
